# GSTT1/GSTM1 deficiency aggravated cisplatin-induced acute kidney injury via ROS-triggered ferroptosis

**DOI:** 10.3389/fimmu.2024.1457230

**Published:** 2024-09-25

**Authors:** Peipei Li, Duopin Li, Yanfang Lu, Shaokang Pan, Fei Cheng, Shen Li, Xiaonan Zhang, Jinling Huo, Dongwei Liu, Zhangsuo Liu

**Affiliations:** ^1^ Department of Nephrology, The First Affiliated Hospital of Zhengzhou University, Zhengzhou, China; ^2^ Research Institute of Nephrology, Zhengzhou University, Zhengzhou, China; ^3^ Henan Province Research Center For Kidney Disease, Zhengzhou, China; ^4^ Key Laboratory of Precision Diagnosis and Treatment for Chronic Kidney Disease in Henan Province, Zhengzhou, China; ^5^ Department of Neurology, The First Affiliated Hospital of Zhengzhou University, Zhengzhou, Henan, China

**Keywords:** AKI, cisplatin, GSTT1, GSTM1, ROS, ferroptosis

## Abstract

**Introduction:**

Cisplatin is a widely used chemotherapeutic agent prescribed to treat solid tumors. However, its clinical application is limited because of cisplatin- induced nephrotoxicity. A known complication of cisplatin is acute kidney injury (AKI). Deletion polymorphisms of GSTM1 and GSTT1, members of the glutathione S-transferase family, are common in humans and are presumed to be associated with various kidney diseases. However, the specific roles and mechanisms of GSTM1 and GSTT1 in cisplatin induced AKI remain unclear.

**Methods:**

To investigate the roles of GSTM1 and GSTT1 in cisplatin-induced AKI, we generated GSTM1 and GSTT1 knockout mice using CRISPR-Cas9 technology and assessed their kidney function under normal physiological conditions and cisplatin treatment. Using ELISA kits, we measured the levels of oxidative DNA and protein damage, along with MDA, SOD, GSH, and the GSH/GSSG ratio in wild-type and GSTM1/GSTT1 knockout mice following cisplatin treatment. Additionally, oxidative stress levels and the expression of ferroptosis-related proteins in kidney tissues were examined through Western blotting, qPCR, immunohistochemistry, and immunofluorescence techniques.

**Results:**

Here, we found that GSTT1 and GSTM1 were downregulated in the renal tubular cells of AKI patients and cisplatin-treated mice. Compared with WT mice, *Gstm1/Gstt1*-DKO mice were phenotypically normal but developed more severe kidney dysfunction and exhibited increased ROS levels and severe ferroptosis after injecting cisplatin.

**Discussion:**

Our study revealed that GSTM1 and GSTT1 can protect renal tubular cells against cisplatin-induced nephrotoxicity and ferroptosis, and genetic screening for GSTM1 and GSTT1 polymorphisms can help determine a standard cisplatin dose for cancer patients undergoing chemotherapy.

## Introduction

Acute kidney injury (AKI) is a clinical syndrome, which presents with a rapid decline in kidney function. It is pathologically characterized by tubular necrosis, which leads to high mortality (1, 2). Annually, AKI leads to approximately 1.7 million deaths worldwide (3). Moreover, AKI can progress to chronic kidney disease (CKD) and end-stage renal disease (ESRD). Currently, there is no specific treatment strategy other than supportive care and dialysis (4). Therefore, clarifying the molecular mechanism underlying AKI pathogenesis is helpful for the early diagnosis and targeted treatment of AKI.

Cisplatin is a widely used chemotherapeutic agent known for its nephrotoxic effects. It induces acute kidney injury (AKI) through several mechanisms, including the generation of reactive oxygen species (ROS), inflammation, DNA damage, and programmed necrosis of renal tubular cells (5). Cisplatin induces the production of reactive oxygen species (ROS), leading to oxidative stress and damage to cellular components, including lipids, proteins, and DNA. The imbalance between ROS production and the antioxidant defense system results in significant cellular injury (6). Additionally, cisplatin treatment triggers a robust inflammatory response in the kidneys, including the activation of various inflammatory cytokines and chemokines, recruitment of immune cells, and further exacerbation of tissue damage (7). Furthermore, cisplatin forms DNA adducts, leading to DNA damage and activation of the DNA damage response, resulting in cell cycle arrest and apoptosis, contributing to renal injury (8).

Glutathione S-transferases (GSTs) are a superfamily of phase II detoxification enzymes, which can eliminate oxygen radicals, lipid peroxidation products, and toxins by catalyzing electrophilic compounds coupled to glutathione (GSH). It is generally considered to be a detoxification reaction (9) (10). They also contribute to antioxidant defense by detoxifying ROS and other reactive intermediates, maintaining redox balance, and protecting against oxidative stress (11, 12). Human GST genes are divided into four major subfamilies, named GSTA, GSTM, GSTT, and GSTP (13). GSTs are highly expressed in most tissues, whereas GSTM1 and GSTT1 are mainly expressed in the liver and kidneys (14, 15). GSTM1 and GSTT1 are polymorphisms with null genotypes for each gene, with approximately 10% to 65% prevalence in the general population (16–19). Deletion or null genotypes of GSTM1 and GSTT1 can significantly increase the risk of ESRD (20–22). Recently, a study showed that deletion of GSTM1 exacerbates kidney injury and intensifies oxidative stress and inflammation in the model mice of CKD (23). These findings suggest that GSTM1 and GSTT1 can protect the kidneys; however, it is still unclear whether GSTM1 and GSTT1 are involved in the development and progression of AKI.

Ferroptosis is a new modality of cell death discovered by Dixon in 2012. Unlike apoptosis and necrosis, ferroptosis is characterized by an iron-dependent cell death pattern accompanied by lipid peroxidation (24, 25). The solute carrier family 7 member 11 (SLC7A11) and glutathione peroxidase 4 (GPX4) are inhibited during ferroptosis, decreasing GSH levels and increasing ROS levels (26, 27). Therefore, ROS play an integral role in the occurrence and progression of ferroptosis (28, 29). Recent studies have found that the occurrence of ferroptosis in renal tubular cells is an important pathological change in AKI, and promoting ferroptosis of renal tubular epithelial cells can expedite the progression of AKI (30). On the contrary, inhibition of ferroptosis in renal tubular epithelial cells can significantly protect against AKI (31–34). However, the specific mechanism of ferroptosis in renal tubular epithelial cells in AKI necessitates further studies.

In this study, we found that GSTT1 and GSTM1 were downregulated in the renal tubules of patients with AKI and cisplatin-treated mice. Compared with WT mice, *Gstm1/Gstt1-*DKO mice were phenotypically normal but developed more severe kidney dysfunction and higher ROS levels after receiving cisplatin, leading to ferroptosis in renal tubular cells and aggravating acute kidney injury. In conclusion, our study revealed that GSTM1 and GSTT1 can protect renal tubular cells against cisplatin-induced AKI and ferroptosis.

## Results

### The expression and localization of GSTM1 and GSTT1

**Figure 1:**
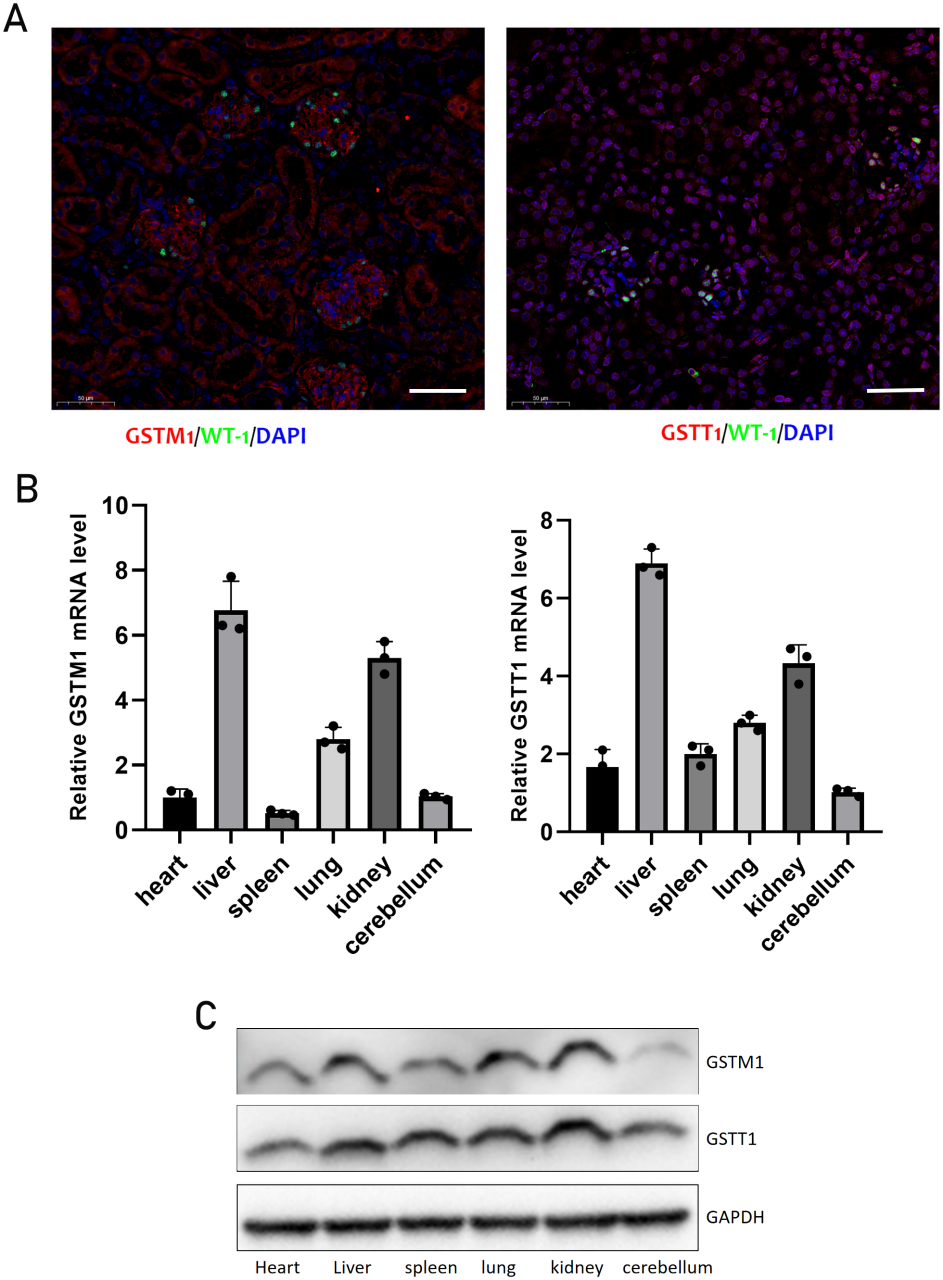
The expression and localization of GSTM1 and GSTT1 **(A)** Immunofluorescence staining of GSTT1 and GSTM1 in renal tissue from heathy mice. **(B)** qPCR analysis of Gstm1 and Gstt1 in different tissues such as heart, liver, spleen, lung, kidney and cerebellum in WT mice. **(C)** Protein expression levels of Gstm1 and Gstt1 in different tissues in WT mice. Scale bar = 50μm. n=3.

Genetic variations in GSTM1 and GSTT1 have been linked to kidney diseases. We studied their expression in different organs of mice and found that they are most highly expressed in the kidneys and liver, reflecting their detoxification and antioxidant roles (**Figures 1B, C**). To elucidate the precise localization of GSTM1 and GSTT1 in the kidney, we utilized paraffin-embedded kidney tissues from 2-month-old mice and conducted immunofluorescence staining. Our findings indicated robust expression of GSTM1 and GSTT1 in both glomerular and tubular regions, with GSTT1 predominantly localized in the cytosol and nucleus, and GSTM1 exclusively expressed in the cytoplasm (**Figure 1A**).

### The expression levels of both GSTM1 and GSTT1 were significantly reduced in patients with AKI

**Figure 2:**
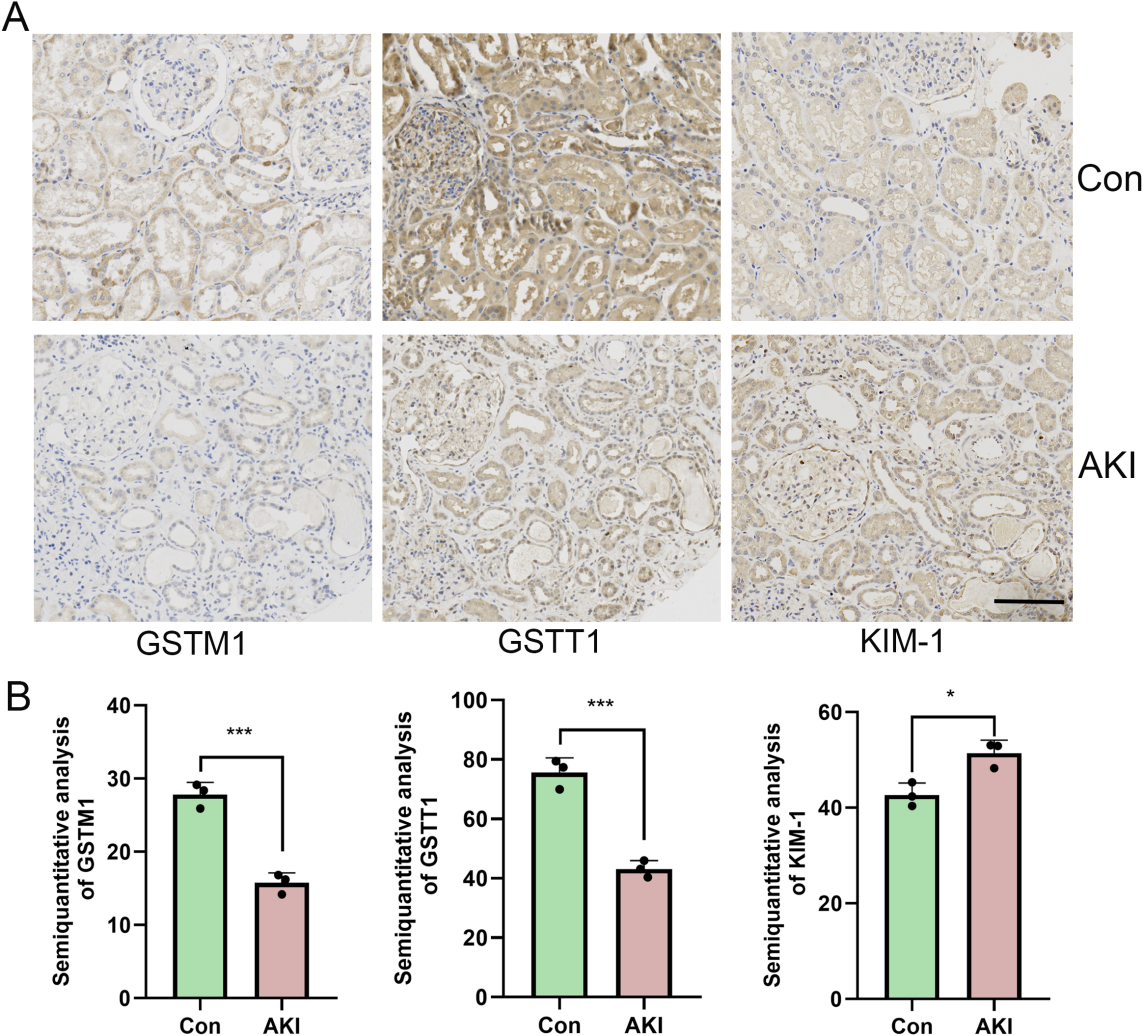
The expression of both GSTM1 and GSTT1 were significantly reduced in AKI patients **(A)** Immunohistochemical staining results of GSTT1, GSTM1 and KIM-1 in renal puncture tissue of AKI patients and para-cancerous control tissue. **(B)** Quantitative analysis of GSTM1, GSTT1 and KIM-1 immunohistochemical staining. The data are presented as the mean ± SD, *P<0.05, **P<0.01, ***P<0.001, n=3. Scale bar =100μm.

Kidney biopsy samples from patients with acute kidney injury (AKI) and kidney para-cancer tissues from patients with kidney cancer (the kidney specimens without histopathological lesions were served as normal controls) were collected for immunohistochemical staining. The results revealed a significant increase in the expression levels of KIM-1, a biomarker of renal tubular injury during AKI, whereas the expression levels of GSTT1 and GSTM1 were markedly decreased (**Figure 2**).

### The expression levels of both GSTM1 and GSTT1 were significantly reduced in mice with cisplatin-induced AKI

**Figure 3:**
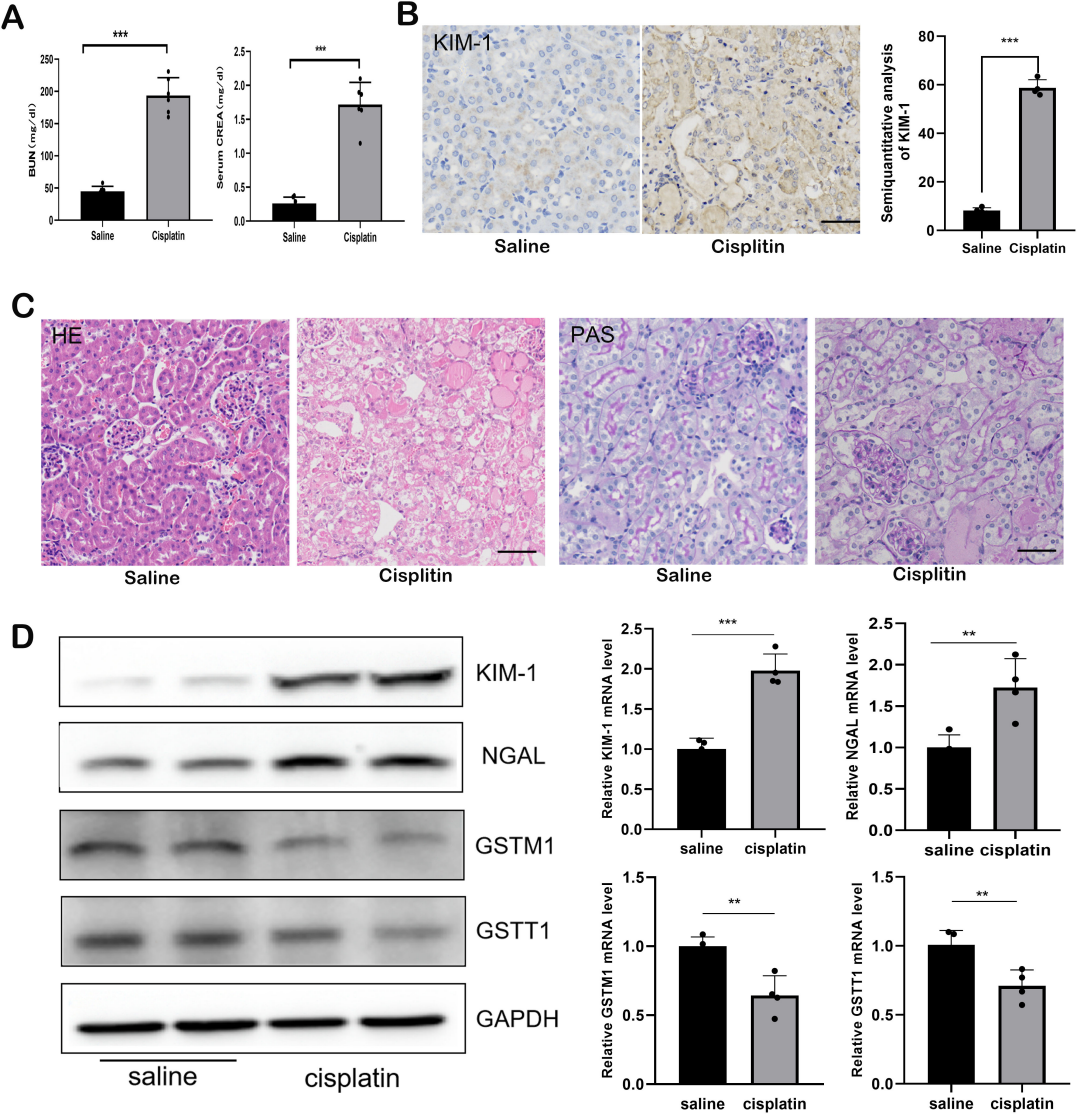
The expression of both GSTM1 and GSTT1 were significantly reduced in mice with cisplatin-induced AKI. **(A)** the BUN and CERA levels after 3 days of cisplatin and saline injection. n=6. **(B)** the expression of kim-1 was detected by immunohistochemical staining after 3days of Cisplatin and saline injection. **(C)** Kidney histology as shown by periodic acid–Schiff (PAS) and HE staining after 3 days of cisplatin and saline injection **(D)** Western blots and qPCR analysis of Kim-1, NGAL, GSTM1 and GSTT1 in renal tissue from these two groups (KIM-1 and NGAL was significantly upregulated, whereas the expression of GSTM1 and GSTT1 were significantly reduced). The data are presented as the mean ± SD, *P<0.05, **P<0.01, ***P<0.001, n≥3. Scale bar =40μm.

Mice received intraperitoneal injections of saline or cisplatin, and the expression levels of GSTT1 and GSTM1 were measured. Compared to the WT + saline group, blood urea nitrogen (BUN) and creatinine (CREA) levels were significantly elevated in the WT + Cis group (**Figure 3A**). Immunohistochemical staining and Western blotting demonstrated a significant increase in the expression levels of KIM-1 and NGAL (**Figures 3B, D**). Histological staining with hematoxylin and eosin (HE) and periodic acid-Schiff (PAS) revealed marked dilation of renal tubules and protein tubule structure in mice receiving cisplatin (**Figure 3C**), accompanied by a significant reduction in GSTM1 and GSTT1 expression (**Figure 3D**).

### Construction of GSTM1 and GSTT1 knockout mice

**Figure 4:**
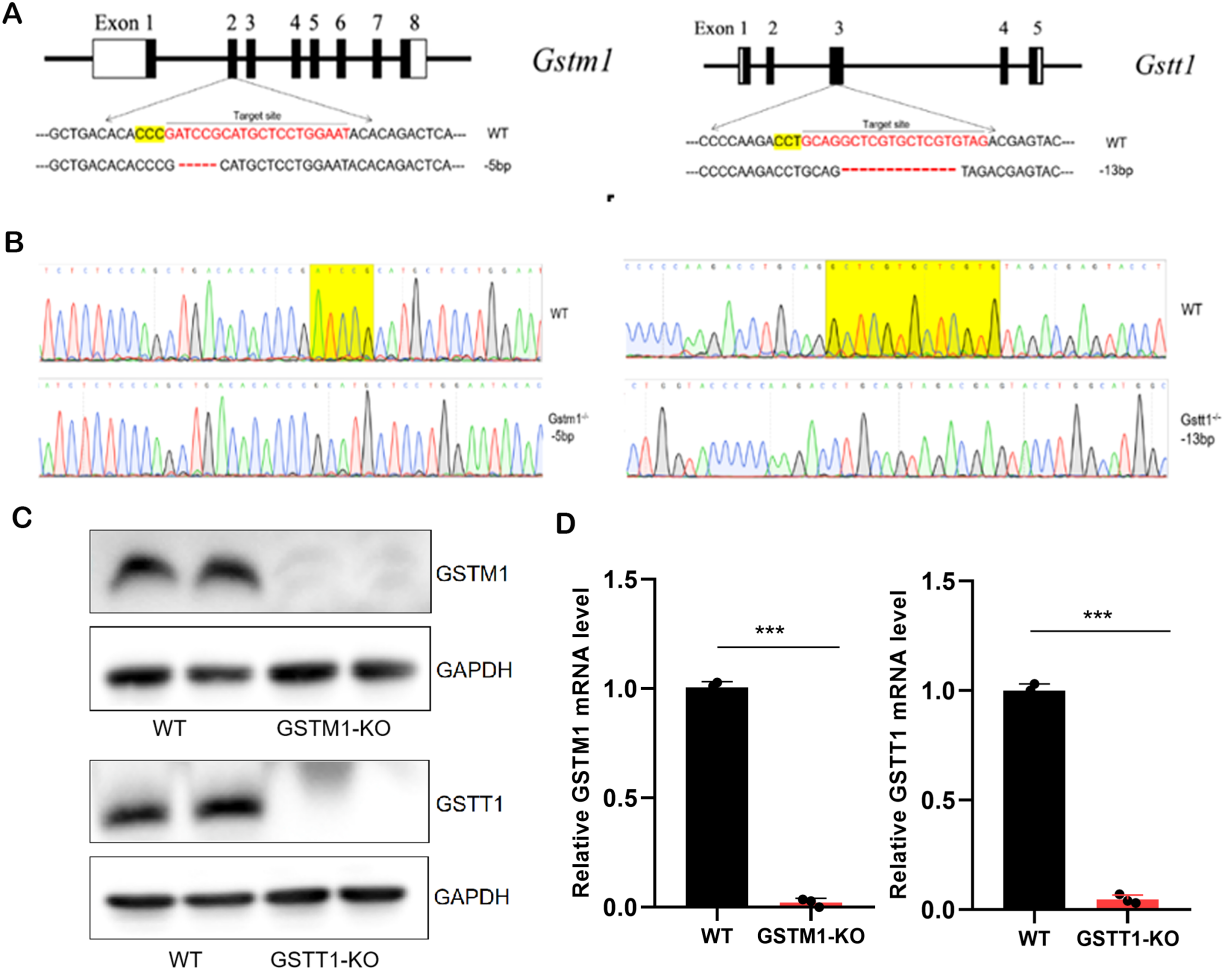
Generation of GSTM1-KO and GSTT1-KO mice **(A)** Schematic overview of generation of GSTT1 and GSTM1 knockout mice using CRISPR/Cas9 system. **(B)** Sequence of WT, GSTM1-KO, and GGSTT1-KO mice. **(C)** Protein expression levels of Gstm1 and Gstt1 in the kidney of WT, GSTM1-KO, and GSTT1-KO mice. **(D)** mRNA expression levels of Gstm1 and Gstt1 in the kidney of WT, GSTM1-KO, and GSTT1-KO mice. The data are presented as the mean ± SD, *P<0.5, **P<0.01, ***P<0.001, n≥3.

**Figure 5:**
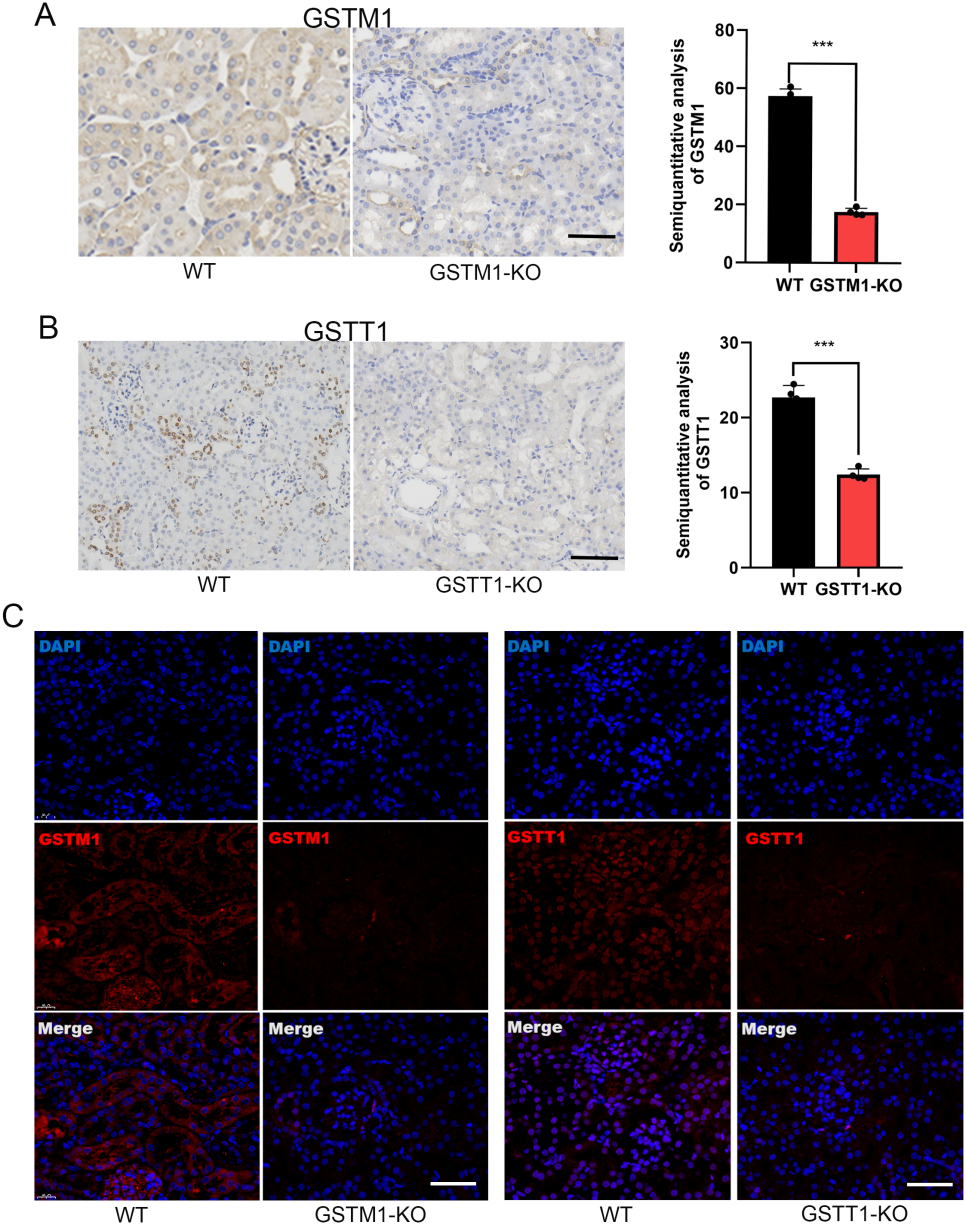
Generation of GSTM1-KO and GSTT1-KO mice. Immunohistochemistry **(A, B)** and immunofluorescence staining **(C)** also showed that the expression levels of GSTM1 and GSTT1 were significantly reduced in GSTM1-KO and GSTT1-KO mice. The data are presented as the mean ± SD, *P<0.5, **P<0.01, ***P<0.001, n=4. Scale bar =40μm.

To further investigate the mechanism by which GSTM1 and GSTT1 are involved in the pathogenesis of acute kidney injury (AKI), we successfully constructed mice with GSTM1 and GSTT1 knockout (GSTM1-KO and GSTT1-KO) using CRISPR-Cas9 technology. The GSTM1 and GSTT1 genes are highly homologous between humans and mice. GSTM1 is located on chromosome 3, spanning 5724 bp and comprising 8 exons that encode 244 amino acids. GSTT1 is located on chromosome 10, spanning 14,772 bp and consisting of 5 exons encoding 190 amino acids. Exon 2 of GSTM1 (GATCCGCATGCTCCTGGAAT) and exon 3 of GSTT1 (GCAGGCTCGTGTAG) were selected as specific targets for knockout (**Figure 4A**). DNA was extracted from mouse tails 7 days after birth, and target bands were amplified using PCR. Due to the small size of the knockout gene fragments (5 bp and 13 bp), conventional agarose gel electrophoresis could not differentiate homozygous, heterozygous, and wild-type genotypes. Therefore, PCR amplification products were sent for gene sequencing, and comparison with wild-type sequences confirmed the successful deletion of the target bases in GSTM1 and GSTT1 (**Figure 4B**). We analyzed mRNA and protein levels to validate the knockout efficiency. Kidney tissues from 2-month-old knockout and wild-type mice were dissected for protein and RNA extraction. Western blotting and real-time PCR showed significantly reduced mRNA and protein expression levels of GSTM1 and GSTT1 in the kidneys of knockout mice compared to wild-type mice (**Figures 4C, D**). Immunohistochemistry and immunofluorescence staining also demonstrated significantly reduced expression levels of GSTM1 and GSTT1 (**Figures 5A–C**), confirming the successful construction of GSTM1-KO and GSTT1-KO mice models.

### Normal kidney function in GSTM1-KO, GSTT1-KO, and Gstm1/Gstt1-DKO mice

**Figure 6:**
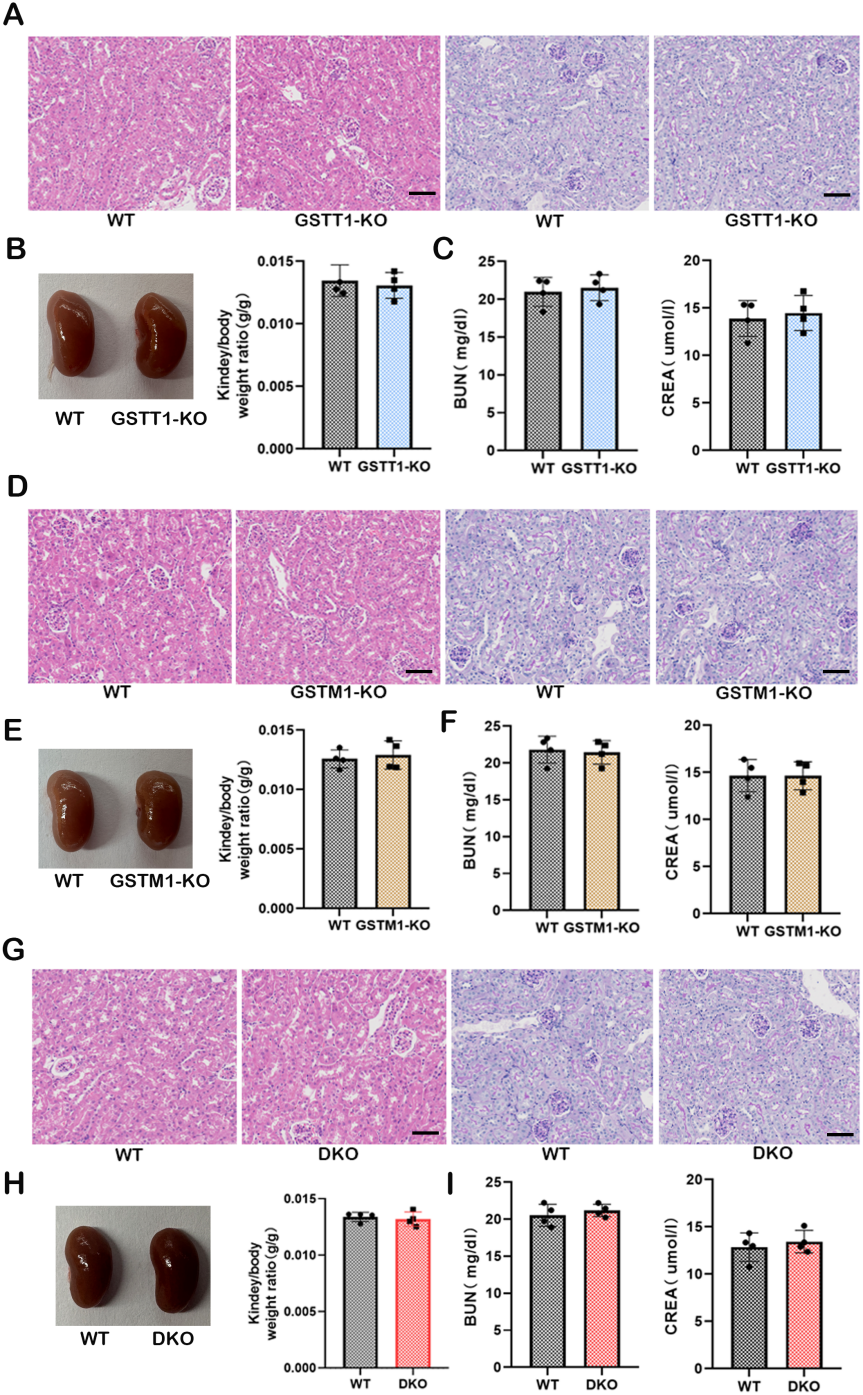
Normal kidney function in GSTM1-KO, GSTT1-KO, and Gstm1/Gstt1-DKO mice. **(A)** HE and PAS staining showed that the structure and morphology of kidney in GSTT1-KO mice was normal. **(B)** There was no difference in renal appearance and renal weight ratio between WT and GSTT1-KO mice. **(C)** BUN and CREA showed that the deletion of GSTT1 did not affect renal function in GSTT1-KO mice. **(D)** HE and PAS staining showed that the structure and morphology of kidney in GSTM1-KO mice was normal. **(E)** There was no difference in renal appearance and renal weight ratio between WT and GSTM1-KO mice. **(F)** BUN and CREA showed that the deletion of GSTM1 did not affect renal function in GSTM1-KO mice. **(G)** HE and PAS staining showed that the structure and morphology of kidney in DKO mice was normal. **(H)** There was no difference in renal appearance and renal weight ratio between WT and DKO mice. **(I)** BUN and CREA showed that the deletion of GSTT1 and GSTM1 did not affect renal function in DKO mice. Scale bar =60μm. The data are presented as the mean ± SD, *P<0.5, **P<0.01, ***P<0.001, n=4.

PAS and HE staining were performed on kidney tissues from 2-month-old *GSTM1-KO*, *GSTT1-KO*, and WT mice to investigate whether the absence of Gstm1 or Gstt1 impairs kidney function and morphology. The results revealed that the kidney structure and morphology were normal in *GSTT1-KO* mice (**Figure 6A**), with no significant differences in kidney-to-body weight ratio (**Figure 6B**), BUN, and CREA levels (**Figure 6C**) compared to wild-type mice. Similar findings were observed in GSTM1-KO mice (**Figures 6D–F**). These results demonstrated that there is no significant difference in the appearance of *GSTM1-KO* or *GSTT1-KO* mice compared to WT mice.

The GSTM1/GSTT1 double-genotype deletion is frequently observed in the general population and is associated with an increased risk of various diseases, including cancer and kidney diseases (28–30). These proteins belong to the GST family, and due to their structural similarity and gene polymorphisms within the GST family, it has been hypothesized that these proteins have similar roles. Based on these considerations and previous reports, we aimed to assess this hypothesis by generating a double-knockout mice model. Therefore, we bred *GSTM1-KO* and *GSTT1-KO* mice together to obtain GSTM1/GSTT1 double-knockout mice (*Gstm1/Gstt11*-DKO). The structure and morphology of the kidneys remained intact in DKO mice (**Figure 6G**). In addition, there were no significant differences in kidney-to-body weight ratio (**Figure 6H**), BUN, and CREA (**Figure 6I**) between DKO mice and wild-type mice.

### Deletion of GSTM1 and GSTT1 aggravated cisplatin-induced AKI in mice

**Figure 7:**
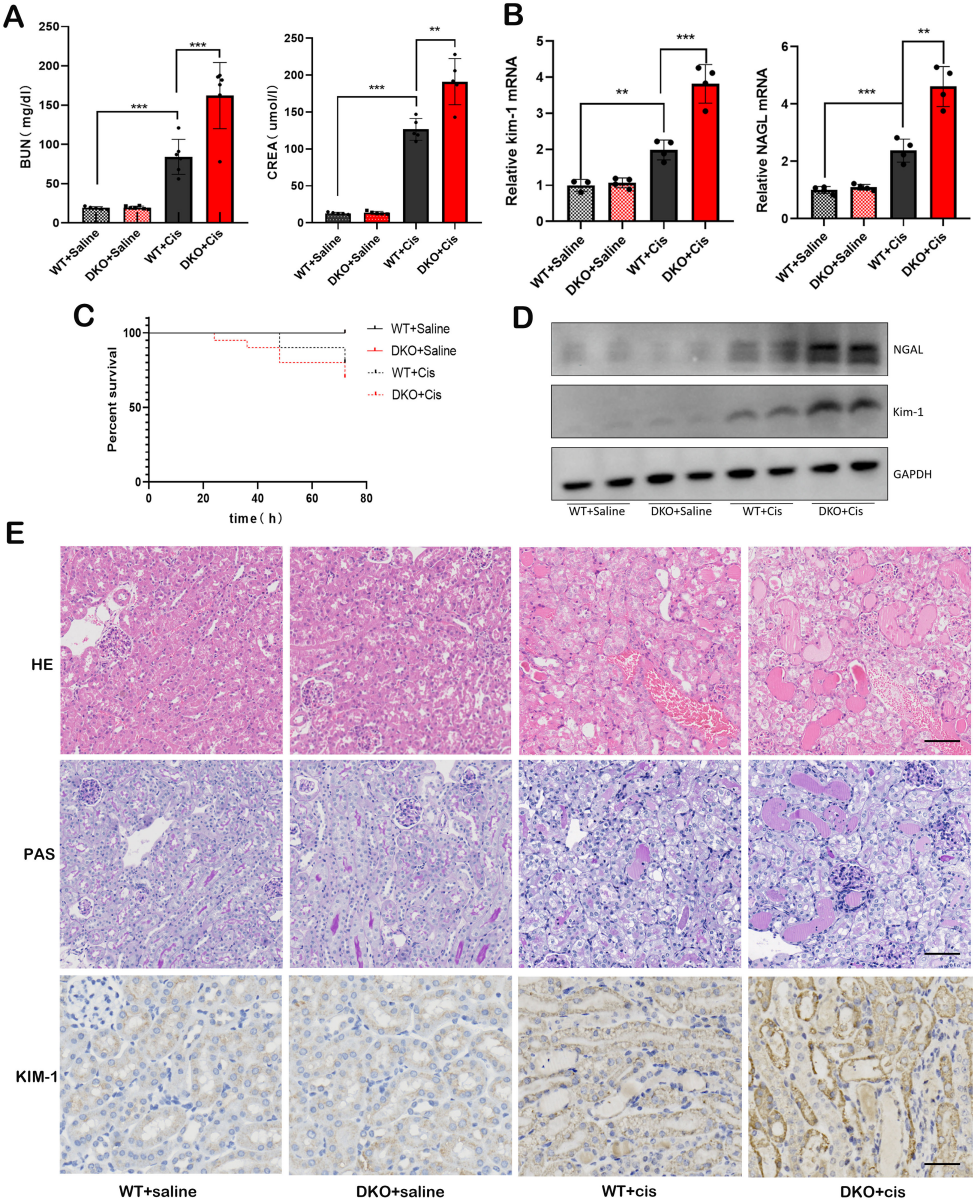
Deletion of GSTM1 and GSTT1 aggravated cisplatin-induced AKI in mice. **(A)** the BUN and CREA levels of four groups of wild type and DKO mice at 3days after cisplatin injection. **(B)** the mRNA and **(D)** protein expression levels of Kim-1 and NGAL were significantly increased in DKO mice after cisplatin injection. **(C)** the survival curve analysis of WT and DKO mice after cisplatin injection. **(E)** HE, PAS (Scale bar =50μm) and immunohistochemical (Scale bar =30μm) staining showed that after cisplatin injection, renal tubules of DKO mice were dilated and a large number of protein tubules appeared, and KIM-1 expression was significantly increased compared with WT mice. The data are presented as the mean ± SD, *P<0.5, **P<0.01, ***P<0.001, n=4.

Since the expression of GSTM1 and GSTT1 is downregulated in the kidney tissues of mice and patients with AKI, we hypothesized that GSTM1 and GSTT1 play an important role in the development of AKI. Mice were injected intraperitoneally with cisplatin to induce AKI, and the control mice received an equivalent amount of saline. Compared with the WT + Saline group, the levels of BUN, CREA, KIM-1, and NGAL, and the severity of renal tubular damage significantly increased in the WT + Cis group (**Figures 7A, B, E**), indicating the successful induction of cisplatin-induced mice model of AKI. However, the serum levels of BUN, CREA, KIM-1, and NGAL and the severity of renal tubular damage showed a greater increase in the DKO + Cis group compared to the WT + Cis group (**Figures 7A, B, D, E**), suggesting that co-deletion of GSTM1 and GSTT1 exacerbated cisplatin-induced AKI. Additionally, survival analysis showed a higher mortality rate in DKO mice after treatment with cisplatin (**Figure 7C**). These results suggest that simultaneous deletion of GSTM1 and GSTT1 aggravated cisplatin-induced AKI in mice.

### Following cisplatin injection, DKO mice exhibited a significantly elevated level of oxidative stress compared to WT mice

**Figure 8:**
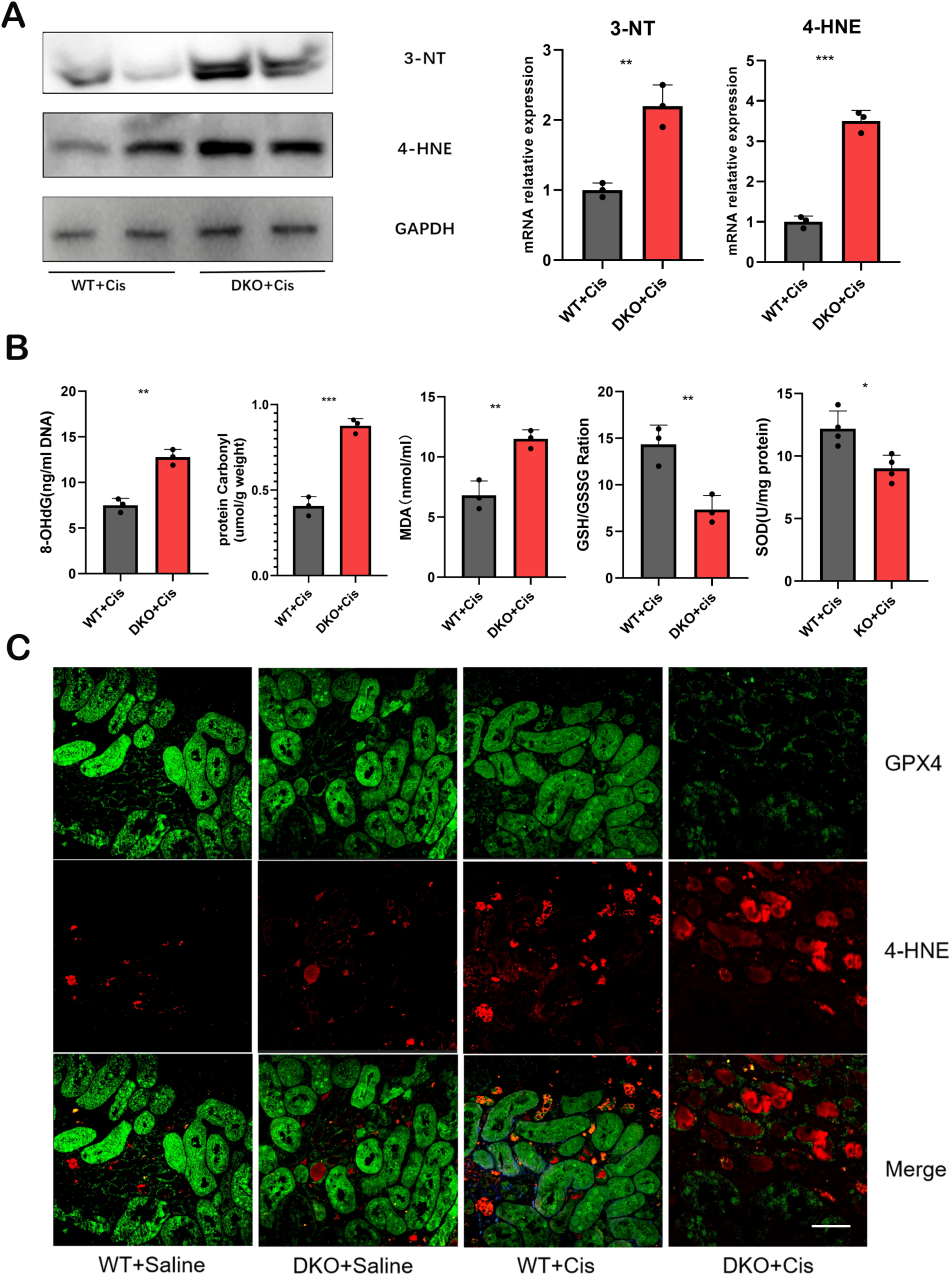
The level of oxidative stress was significantly increased in DKO mice after cisplatin injection. **(A)** the mRNA and protein expression levels of 4-HNE and 3-NT were significantly increased in DKO mice compared with WT mice after cisplatin injection. **(B)** the 8-OHdG, protein carbonyl, MDA,GSH/GSSG and SOD expression level in WT and DKO mice after cisplatin injection. **(C)** Representative immunofluorescence staining of 4-HNE and GPX4 in WT and DKO mice after cisplatin injection. Scale bar =40μm. The data are presented as the mean ± SD, *P<0.5, **P<0.01, ***P<0.001, n≥3.

Under normal physiological conditions, glutathione (GSH) primarily exists in its reduced form, with GSH oxidized to oxidized glutathione (GSSG). GSSG is subsequently reduced back to GSH by glutathione reductase, maintaining cellular redox equilibrium. Thus, the GSH/GSSG redox system is pivotal for intracellular antioxidant capacity. ELISA revealed a significant decrease in the GSH/GSSG ratio in cisplatin-induced DKO mice (**Figure 8B**), indicating increased ROS production and impaired redox homeostasis after cisplatin exposure. The levels of malondialdehyde (MDA), a marker of lipid peroxidation, were notably elevated in these mice (**Figure 8B**). Superoxide dismutase (SOD) is an enzyme crucial for scavenging O2- and converting it into H_2_O_2_. H_2_O_2_ is further detoxified by peroxidases, catalase, and glutathione peroxidase to attenuate oxidative stress. SOD levels were significantly reduced (**Figure 8B**), while the mRNA and protein expression levels of the oxidative stress markers, 4-hydroxynonenal (4-HNE) and 3-nitrotyrosine (3-NT), were markedly increased in DKO mice exposed to cisplatin (**Figures 8A, C**).

To illustrate the imbalance between reactive oxygen species and antioxidant systems, we evaluated oxidative damage products and detected MDA and SOD levels and GSH/GSSG ratio. 8-hydroxy-2-deoxyguanosine (8-OHdG) is an oxidative product formed from cisplatin-induced oxidative damage to nuclear DNA. It is a common clinical and experimental biomarker used to assess oxidative damage to DNA. Protein carbonylation includes oxidation-induced damage to amino acid side chains, altering protein structure and function. Accumulation of carbonyl products can help evaluate organismal oxidation levels. Our results demonstrated significantly elevated levels of 8-OHdG and protein carbonylation in cisplatin-induced DKO mice compared to WT mice (**Figure 8B**), suggesting that the deletion of GSTM1 and GSTT1 reduces the antioxidant capacity of mice.

### Following cisplatin injection, DKO mice exhibited more pronounced ferroptosis compared to WT mice

**Figure 9:**
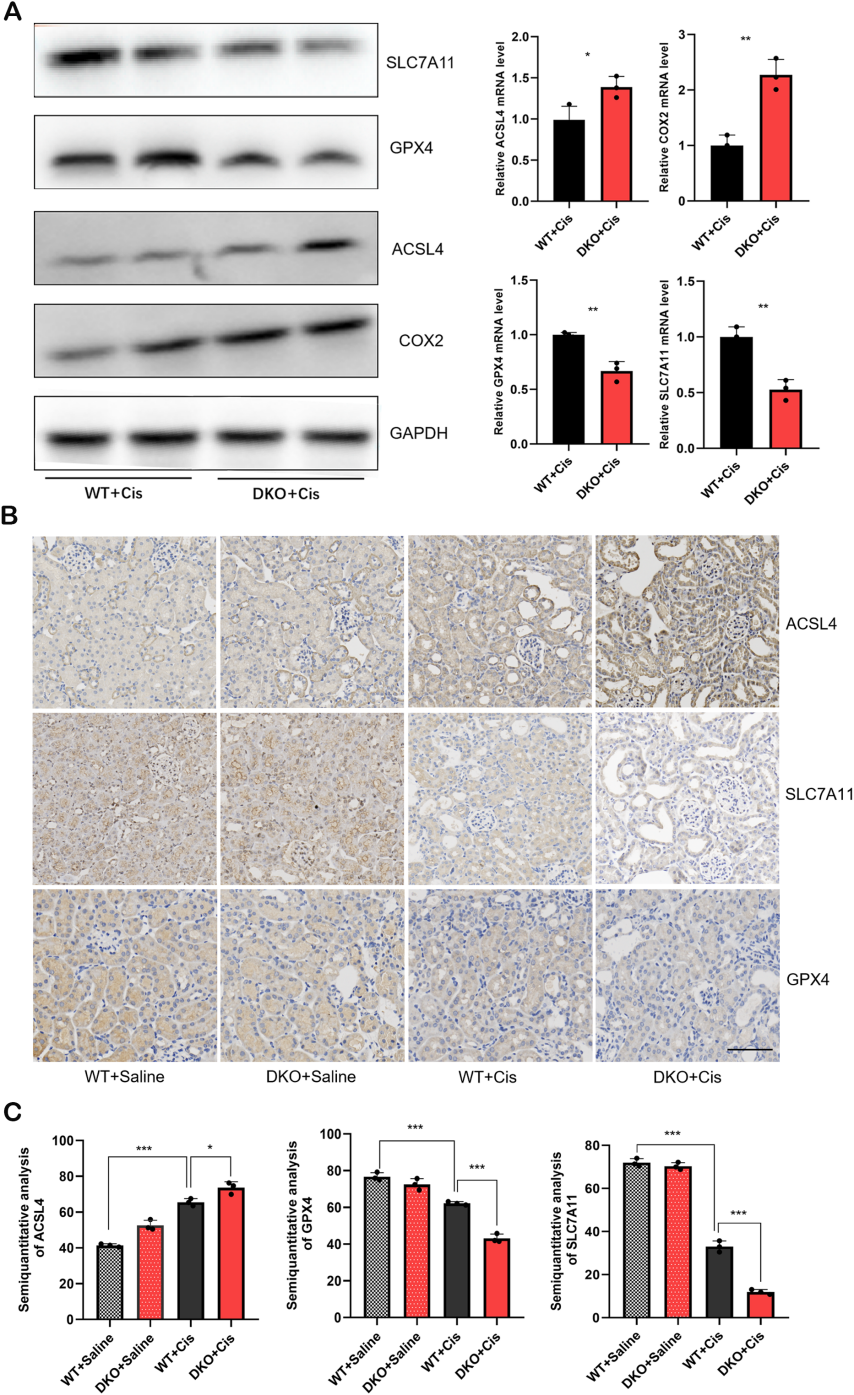
DKO mice showed more severe ferroptosis than WT mice after cisplatin injection **(A)** the mRNA and protein levels of ferroptosis related proteins in WT and DKO mice after cisplatin injection. **(B)** Immunohistochemical staining images of ferroptosis related proteins in WT and DKO mice after cisplatin injection. Scale bar =60μm. **(C)** Quantitative analysis of ACSL4, SLC7A11 and GPX4 immunohistochemical staining. The data are presented as the mean ± SD, *P<0.5, **P<0.01, ***P<0.001, n≥3.

Our previous study has confirmed that deletion of the GSTM1 and GSTT1 can reduce the antioxidant capacity of mice, rendering them more susceptible to cisplatin-induced oxidative damage. Specifically, the GSH/GSSG ratio significantly reduces after deleting these genes, and the expression of MDA, 3-NT, and 4-HNE markedly increases after deleting these genes. Based on these findings, we hypothesized that GSTT1 and GSTM1 knockout can primarily affect ferroptosis in renal tubular cells. To assess this hypothesis, we analyzed the expression levels of ferroptosis-related proteins in DKO mice and WT mice exposed to cisplatin. The results revealed significantly lower expression levels of SLC7A11 and GPX4 and higher expression levels of ACSL4 and COX2 in DKO mice compared to WT mice (**Figure 9A**). Immunohistochemical staining and immunofluorescence also validated these findings (**Figures 8C**, **9B, C**), indicating that GSTT1 and GSTM1 knockout profoundly disrupts the oxidative equilibrium in mice, aggravating lipid peroxidation and ferroptosis.

### Fer-1 and NAC can alleviate cisplatin-induced AKI in *Gstm1/Gstt1*-DKO mice

**Figure 10:**
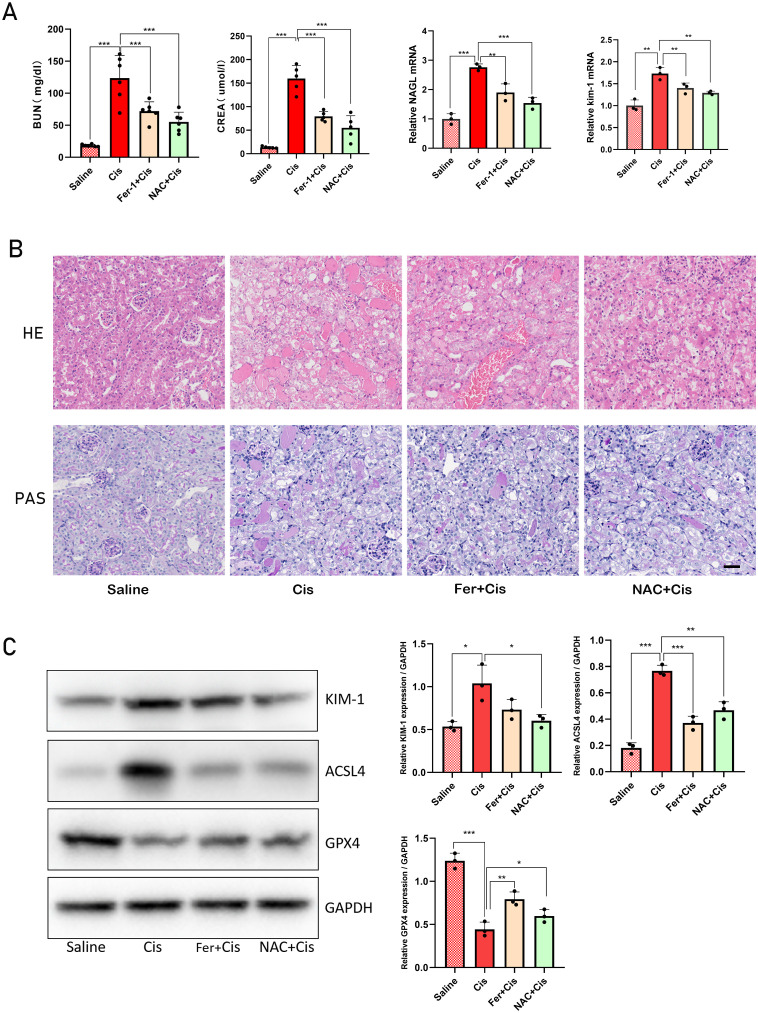
Fer-1 and NAC can alleviate cisplatin-induced AKI in Gstm1/Gstt1-DKO mice **(A)** the BUN, CREA, NGAL and kim-1 levels were measured in four groups of DKO mice treated with different treatments. **(B)** HE and PAS staining images of four groups of DKO mice treated with different treatments. **(C)** Protein expression levels of KIM-1, ACSL4 and GPX4 in different tissues in four groups of DKO mice treated with different treatments. Scale bar =50μm. The data are presented as the mean ± SD, *P<0.5, **P<0.01, ***P<0.001, n≥3.

We conducted a rescue experiment to validate that the primary mode of tubular cell death is ferroptosis in DKO mice exposed to cisplatin. DKO mice received 5 mg/kg of the ferroptosis inhibitor Ferrostatin-1 (Fer-1) and 325 mg/kg of the antioxidant N-Acetyl-L-cysteine (NAC) before administering cisplatin. The experimental groups included DKO, DKO + cisplatin, DKO + cisplatin + Fer-1, and DKO + cisplatin + NAC groups. Compared with cisplatin treatment group, BUN, CREA, KIM-1 and NGAL levels and the severity of renal tubule damage in Fer-1 and NAC pretreatment groups were significantly reduced. Those results demonstrated that Fer-1 and NAC effectively mitigated cisplatin-induced kidney damage in DKO mice (**Figures 10A, B**). In addition, we quantitatively analyzed the expression levels of ferroptosis related genes such as GPX4 and ACSL4, as well as kidney injury marker genes KIM-1 and NGAL to confirm the effectiveness of treatment. The result of WB showed that NAC and Fer-1 pretreatment could significantly reduce cisplatin induced ferroptosis in DKO mice (**Figure 10C**).

## Discussion

AKI is a clinically critical condition characterized by a rapid decline in renal function in the short term. Pre-renal factors (insufficient renal blood flow caused by cardiac surgery (35, 36) and severe dehydration), renal factors (renal lesions caused by nephrotoxic drugs or sepsis) (5, 37, 38), and post-renal factors (urinary tract obstruction by urinary system tumors and stones) are the main causes of AKI (39). Due to the toxic effects of various drugs or foods on the kidneys, AKI caused by nephrotoxic drugs is very common. Cisplatin (CP) is currently one of the most widely used chemotherapy drugs. Cisplatin has a toxic effect on renal tubular epithelial cells; thus, the incidence of AKI in patients receiving cisplatin is as high as 30% to 40, which greatly decreases the anti-tumor use of cisplatin (40, 41). In recent years, many studies have been conducted on the pathogenesis of cisplatin-induced AKI, but it is still unclear. There is still a lack of safe and effective interventions that can adequately treat or prevent cisplatin-induced AKI. Therefore, it is important to unravel the pathogenesis of cisplatin-induced AKI and develop new therapeutic means to effectively improve AKI in patients receiving cisplatin.

Glutathione S-transferases (GSTs) are a superfamily of phase II detoxification enzymes that protect cells by catalyzing many conjugation processes of hazardous chemicals to reduce glutathione (GSH). Deletion polymorphisms of genes encoding GSTM1 and GSTT1 are widely found in humans. They are clinically of high significance as they affect the metabolism of exogenous active substances and disease susceptibility among different individuals (42–45). The relationship between these enzymes and kidney function has been a topic of considerable interest, given the kidneys’ vital role in filtering and detoxifying blood. Several studies have explored the correlation between GSTT1 and GSTM1 polymorphisms and susceptibility to various renal diseases, including acute kidney injury (AKI) and chronic kidney disease (CKD) (21, 46–48). The presence or absence of these genes can influence an individual’s ability to detoxify harmful substances, thereby affecting renal function. Our previous studies have also shown that the loss of GSTT1 and GSTM1 is associated with cisplatin-induced ototoxicity (49). In this study, we found that the expression of both GSTM1 and GSTT1 was significantly reduced in patients and mice with AKI. Deletion of GSTM1 and GSTT1 aggravated cisplatin-induced AKI in mice. Therefore, GSTT1 and GSTM1 play a crucial role in maintaining kidney function by reducing oxidative stress and detoxifying harmful substances.

Cisplatin induces the accumulation of lipid peroxidation products in renal tubular epithelial cells, inhibits the antioxidant system, and enhances ROS production, which are the main mechanisms leading to AKI (50). Studies have shown that glutathione consumption can increase intracellular oxidative stress. Inhibition of oxidative stress can ameliorate cisplatin-induced renal tubular epithelial cell damage. Our findings revealed that SOD and GSH/GSSG levels were significantly decreased, and MDA levels were significantly increased in cisplatin-induced DKO mice compared to WT mice. After exposure to cisplatin, 8-OHdG and protein carbonyl levels were significantly higher in DKO mice than in WT mice. In this study, we also found that after exposure to cisplatin, the oxidative stress markers, 4-HNE and 3-NT, were significantly higher in DKO mice compared to WT mice. These results indicated that the deletion of GSTM1 and GSTT1 disrupted the REDOX balance in mice.

Similar to our findings, Chang et al. showed that GSTM1 knockout *in vitro* can upregulate 4-HNE (49). Siems et al. demonstrated that GSTM1 (0) homozygous subjects had higher plasma levels of MDA than subjects with active alleles (51). Moreover, multiple studies have shown that the plasma levels of 4-HNE and MDA are significantly elevated in patients with renal failure and are directly associated with the degree of renal damage (52). Therefore, determining the relationship between GSTMI and GSTT1 genotypes and circulating 4-HNE levels in normal conditions and AKI is a future research focus.

Nrf2 can promote the transcription of certain antioxidant genes. It plays a key role in attenuating the toxicity induced by exogenous compounds. Our study showed that compared with WT mice, the mRNA and protein expression levels of Nrf2 were significantly increased in the DKO mice (data not shown). This finding suggests that the absence of GSTM1 and GSTT1 affects the expression of Nrf2, possibly because its high expression in stress conditions can slow the renal toxicity of cisplatin. In addition, our data showed that under normal physiological conditions, the expression of other GST genes, such as GSTM2 and GSTA4, was increased in GSTT1-KO mice and GSTM1-KO mice (**Supplementary Figure 1**). This upregulation may help explain why GSTM1 KO and GSTT1 KO mice exhibit normal renal function under normal physiological conditions despite the absence of GSTM1 or GSTT1.

Ferroptosis is a unique type of cell death triggered by intracellular accumulation of free iron, which leads to the accumulation of lipid peroxides on the cell membrane (25). In recent years, ferroptosis has been considered a key pathological form of cell death in cisplatin-induced AKI (53, 54). Our research results indicate that Gstm1 and Gstt1 double knockout (DKO) mice show increased susceptibility to cisplatin-induced acute kidney injury (AKI). We observed that cisplatin treatment led to elevated levels of oxidative stress and lipid peroxidation in DKO mice, resulting in more severe ferroptosis. The administration of NAC and Fer-1 effectively mitigated kidney damage and reduced ferroptosis levels in these mice. Gstm1 and Gstt1 are key enzymes involved in the detoxification of reactive oxygen species (ROS) through their roles in the glutathione-S-transferase (GST) pathway, which is crucial for maintaining cellular redox homeostasis and protecting cells from oxidative damage. Loss of Gstm1 and Gstt1 function can lead to increased accumulation of ROS and heightened susceptibility to oxidative stress-induced damage (55, 56). Ferroptosis is characterized by the accumulation of lipid peroxides and ROS, and is tightly regulated by cellular redox homeostasis (57). The loss of Gstm1 and Gstt1 disrupts the balance of ROS, leading to increased lipid peroxidation and triggering ferroptosis. Our findings suggest that the absence of these enzymes exacerbates ROS accumulation, thereby promoting ferroptosis and contributing to kidney injury. This disruption was evident in our study, where cisplatin treated DKO mice had significantly increased levels of oxidative stress and lipid peroxidation, leading to severe ferroptosis. Treatment with NAC and Fer-1, known for their antioxidant and ferroptosis-inhibiting properties, successfully rescued the mice from kidney damage and reduced ferroptosis levels. This suggests that Gstm1 and Gstt1 play critical roles in modulating oxidative stress and ferroptosis in cisplatin-induced AKI. Further studies are warranted to elucidate the precise molecular mechanisms by which Gstm1 and Gstt1 regulate ferroptosis, including investigating their interaction with other ferroptosis regulators such as GPX4 and SLC7A11.

At the same time, we conducted further *in vitro* experiments to evaluate the effect of Fer-1 and NAC on the antitumor activity of cisplatin. We followed the previous approach to treat cancer cell lines and HK2 with cisplatin alone (20μM) or in combination with Fer-1 (0.4µM) or NAC (10 mM) (54, 58, 59). The results showed that the addition of Fer-1 or NAC did not significantly alter the cytotoxic effects of cisplatin on these cancer cells (**Supplementary Figure 2**). The reason low doses of Fer-1 and NAC do not affect the anti-tumor efficacy of cisplatin but can mitigate its nephrotoxicity might be related to differences in cell types. These protective agents may exert distinct mechanisms of action in different types of cells. In renal cells of DKO mice(GSTT1/GSTM1-DKO), cisplatin-induced nephrotoxicity is primarily mediated through oxidative stress and ferroptosis pathways, with Fer-1 acting as a ferroptosis inhibitor and NAC as an antioxidant, effectively alleviating damage caused by these mechanisms (60–62). However, in cancer cells, cisplatin exerts its anti-tumor effects mainly by binding to DNA, causing DNA damage, thereby preventing DNA replication and transcription, and ultimately leading to cell death (63, 64). Therefore, low doses of Fer-1 and NAC can provide renal protection without significantly interfering with the anti-tumor efficacy of cisplatin. Additionally, different cell lines may exhibit varying sensitivities to antioxidant and anti-ferroptosis treatment.

In summary, this study indicated that GSTT1 and GSTM1 can alleviate cisplatin-induced AKI by maintaining oxidative stress balance and reducing ferroptosis in renal tubular cells. Activation of GSTT1 and GSTM1 may provide a new therapeutic strategy for AKI.

## Materials and methods

### Animals and ethics statement

All animal experiments (mice) were approved by the Ethics Committee of The First Affiliated Hospital of Zhengzhou University (Ethics Approval No. ZZU-LAC20201013[09]), and in strict accordance with the standards of the Animal Ethics Committee.

Eight-week-old male GSTT1 KO/GSTM1 KO mice and control littermates, on a CBA/J background, were obtained from Professor Gao Jiangang (Shandong University, Jinan, China) and fed under SPF conditions in the Laboratory Animal Center of Zhengzhou University (Zhengzhou, Henan, China). The mice were kept in an SPF environment with room temperature (21 ± 1°C), humidity (75 ± 5%), and light/dark cycles of 12 hours. The animals were acclimated to the environment for 2 weeks prior to the study and were given free access to standard laboratory feed and water.

### Human kidney biopsy samples

Kidney biopsy samples from patients with acute kidney injury (AKI) and kidney para-cancer tissues from patients with kidney cancer (the kidney specimens without histomorphologic lesions were served as normal controls) were obtained from the First Affiliated Hospital of Zhengzhou University, China. This study was approved by the Institutional Ethics Committee of The First Affiliated Hospital. Informed consent is provided by all participants (Ethics Approval number: 2021-KY-004-02).

### Animal model system

Cisplatin (Sigma Chemical Co, USA) was dissolved with 0.9% physiological saline at 2 mg/ml). AKI was induced by a single intraperitoneal (i.p.) injection of cisplatin (20 mg/kg). Control mice received 0.9% physiological saline only. For ferroptosis inhibition studies, Ferrostatin-1(Fer-1) was administered 45 minutes to 2 hours before the induction of AKI at a dose of 5 mg/kg. For ROS scavenger (Sigma-Aldrich, A9165), N-Acetyl-L-cysteine (NAC) was administered 24 hours before the induction of AKI at a dose of 200mg/kg (59, 65, 66). In this study, 8-week-old male mice were selected for each group, with at least 3 mice in each group.

### Measurement of BUN and CREA

Collect the blood specimen of mice in the 1.5ml EP tube. The room temperature is static for 1-2 hours, 3000rpm 4 °C centrifuged for 15 minutes, collecting serum. The contents of BUN and CREA were detected with BUN (Urea Assay Kit) and CREA (Creatinine Assay kit (sarcosine oxidase)) test kits, which were obtained from Nanjing Jiancheng Bioengineering Institute (Nanjing, China).

### MDA, SOD activity Assay and GSH/GSSG ratio

MDA, SOD levels and GSH/GSSG ratio in renal tissues were measured using the corresponding detection kits in accordance with the manufacturer’s instructions. The working solution was prepared according to the instructions, and the serum was mixed with other reagents in the 96-well plate, then incubated at room temperature for 40 min, and the OD value was detected at the corresponding absorbance. Finally, the corresponding indicators were obtained according to the instructions.

### Measurement of DNA and protein damage

Levels of 8-OHdG, a marker of oxidative DNA damage, were evaluated using a bioassay kit (Mlbio, ml002198) according to the instructions. The kidney tissues were homogenized with PBS and then centrifuged at 4 °C for 15 min at 3000 rpm. Transfer the supernatant diluted 4 times to the ELISA plate. Then, the conjugate reagent were added to samples mixture and incubated for an hour under 37°C. And the samples were successively treated with chromogenic agents A and B at 37°C for 15 min to initiate a color reaction. Finally, stop the color reaction with stop solution. The OD value of the samples were obtained at 450 nm.

Levels of protein carbonyl, an oxidative protein damage marker, were evaluated using the protein carbonyl content determination kit (Mlbio, ml076345) according to the manufacturer’s instructions. Kidney tissues were homogenized on ice with lysis buffer (0.1 g/mL) and centrifuged at 8000 rpm for 10 minutes at room temperature for the protein carbonyl immunoassay. Reagent I (9:1) was mixed with the supernatant and left at room temperature for 15 minutes. After centrifugation at 12000 rpm at room temperature for 10 minutes, the supernatants were transferred into clean tubes containing reagent II for 30 minutes. In order to obtain sediment, the sample mixture was added to reagent III and centrifuged for 10 minutes at 12000 rpm at 4°C. The sediment was then homogenized at 37°C for 15 minutes with reagent V after repeated additions of reagent IV. Centrifuge the sample at 4°C for 10 minutes after re-dissolving the sediment. The OD value of the samples were obtained at 370 nm.

### Renal histology and immunohistochemistry staining

Dislocation of the cervical spine was used to sacrifice the mice. The kidneys of mice were quickly removed and preserved in 4% paraformaldehyde. For further analysis, the tissues were dehydrated, embedded in paraffin, and cut into 3 um-thick slices. Then a variety of staining methods were used on the kidney tissues, including H&E, PAS, and immunohistochemistry. The primary antibodies were as follows: kidney injury molecule -1 (Kim-1, 1:200, ab78494,Abcam), glutathione peroxidase 4 (GPX4, 1:100, ab125066, Abcam), ferritin heavy chain 1 (FTH-1, 1:400, 4393, CST), ACSL4 (1:200, 22401-1-AP, Proteintech), cystine/glutamate antiporter solute carrier family 7 member 11 (SLC7A11, 1:200, 26864-1-AP, Proteintech), GSTT1 (1:200, 15838-1-AP, Proteintech), GSTM1 (1:200, 12412-1-AP, Proteintech) and 4-Hydroxynonenal (4-HNE,1:100,MA5-27570,Thermo Fisher).

### Western blotting

The kidney tissues were homogenized in RIPA lysis buffer (Thermo Scientific, 89901) with PMSF and then lysed on ice for 30 min before centrifuged at 10,000rpm for 30 min at 4°C. The protein content of the supernatant is determined by the BCA method. The samples were treated with SDS loading buffer and heated to 100°C for 5 min after transferring the supernatant to an empty centrifuge tube. Using SDS-PAGE, separate each equivalent sample and then transferred to polyvinylidene fluoride(PVDF) membranes. The PVDF membrane was blocked with 5% skim milk for 1 h at room temperature and then incubated with primary antibodies overnight at 4°C. The next day, after washing with TBST, it was incubated for 1 hour with the HRP-conjugated secondary antibody at room temperature. After washing with TBST, the bands were visualized using an ECL system (Tanon, Shanghai, China). The following primary antibodies were used: Gstm1 (1:2000, Proteintech,12412-1-AP), Gstt1 (1:2000, Proteintech,15838-1-AP). TIM-1(1:1000, Abcam, ab78494), GAPDH (1:1000, Servicebio, GB15004), NGAL (1:1000, Abcam, ab633929), Nrf2 (1:1000, Abcam, ab137550), SLC7A11 (1:1000,Proteintech, 26864-1-AP), GPX4 (1:1000, Abcam, ab125066), ACSL4(1:1000, Proteintech, 22401-1-AP), and FTH1 (rabbit polyclonal, 1:1000, Cell Signaling Technology, 4393).

### Quantitative real-time PCR

The total RNA that was derived from kidney tissues was extracted using TRIzol reagent (Invitrogen, 15596026) according to the guidance provided by the manufacturer. And then mRNA was used for reverse transcription with the PrimeScript RT Reagent Kit (Vazyme, R312-01)to produce cDNA. qPCR was performed using Maxima SYBR Green qPCR Master Mix (Thermo Fisher Scientific) and Biosystems QuantStudio 5 (Thermo Fisher Scientific). Values of the housekeeping gene Gapdh were used to as an internal control, and the relative expression level of target genes were evaluated using the comparative CT formula (ΔΔCt). The primers in this study were designed and synthesized by Sangon Biotech and the sequences are listed in the **Table 1**.

**Table 1:** The primer sequences data.

Gene	Forward Primer (5’ –3’)	Reverse Primer (5’ –3’)
GAPDH	ACCCAGAAGACTGTGGATGG	TTCAGCTCAGG GATGACCTT
GSTT1	TGTGGCATAAGGTGATGTTCC	CTTTGTCCTGG AGGAACTTGTCT
GSTM1	GGACTTTCCCAATCTGCCTTAC	GAACAGCCACAAAG TCAGGGT
Nrf2	GCATAGAGCAGGACATGGAGCAAG	ACTGATGGCAGCG GAGGAAGG
GPX4	CATGCCCGATATGCTGAGTGTGG	TAGCACGGCAGGTC CTTCTCTATC
SLC7A11	CCTCTGACGATGGTGATGCTCTTC	GGTGCTGAATGGG TCCGAGTAAAG
ACSL4	GCTATGACGCCCCTCTTTGT	GAATCGGTGTGTCTG AGGGG
FTH1	TGCCATCAACCGCCAGATCAAC	ATTCAGCCCGCTC TCCCAGTC
KIM-1	GGAAGTAAAGGGGGTAGTGGG	GGAAGTAAAGGG GGTAGTGGG
NGAL	GCCCAG GACTCAACTCAGAA	GACCAGGATGGA GGTGACAT
GSTA4	TGATTGCCGTGGCTCCATTTA	CAACGAGAAAAG CCTCTCCGT
GSTM2	ACACCCGCATACAGTTGGC	TGCTTGCCCAG AAACTCAGAG

### Statistical analysis

All data were expressed as a mean ± SEM, and all tests were performed at least three times. Microsoft Excel and GraphPad Prism 8 software (GraphPad software, San Diego, CA, USA) were used to perform statistical analyses. When comparing two groups, two-tailed, unpaired Student’s t tests were applied to indicate the statistical significance. A p-value less than 0.05 was considered significant difference, and the level of significance was indicated as *p < 0.05, **p < 0.01, and ***p < 0.001.

## Data Availability

The data that support the findings of this study are available from the corresponding author upon reasonable request.

## Supplementary material

Supplementary Figure 1 The mRNA levels of GSTM2 and GSTA4 in WT, GSTM1-KO and GSTT1-KO mice. The data are presented as the mean ± SD, **P*<0.5, ***P*<0.01, ****P*<0.001, n=3.

Supplementary Figure 2 The HK2, colon and lung cancer cell lines were pretreated with Fer-1/NAC and then treated with cisplatin for 24 h. Cell survival rate was detected by CCK8 assay. The data are presented as the mean ± SD, *P<0.5, **P<0.01, ***P<0.001, n=4.

Supplementary Figure 3 The mRNA levels of ITGAD, NLE1 and PODN in WT and GSTT1-KO mice. The data are presented as the mean ± SD, *P<0.5, **P<0.01, ***P<0.001, n=3.

Supplementary Figure 4 The mRNA levels of GSTM6, GSTM3 and GSTM2 in WT and GSTM1-KO mice. The data are presented as the mean ± SD, *P<0.5, **P<0.01, ***P<0.001, n=3.
