# Supplementary material for: GSTT1/GSTM1 deficiency aggravated cisplatin-induced acute kidney injury via ROS-triggered ferroptosis
Source: Front Immunol. 2024 Sep 25;15:1457230. doi: 10.3389/fimmu.2024.1457230 (PMC11461197; doi:10.3389/fimmu.2024.1457230)
Supplement: Supplementary file 1 [file DataSheet1.pdf]

	Age(years)	gender	Time to elevated creatinine	Creatinine(μmol/L)	eGFR(ml/min/1.73m^2)
Control1	36	male	-	69	116.094
Control2	40	male	-	80	105.753
Control3	32	female	-	53	121.024
AKI1	62	male	2 days	514	9.56
AKI2	39	male	4 days	916	5.549
AKI3	59	female	7 days	1178	2.7
