# Supplementary figures and images for: GSTT1/GSTM1 deficiency aggravated cisplatin-induced acute kidney injury via ROS-triggered ferroptosis
Source: Front Immunol. 2024 Sep 25;15:1457230. doi: 10.3389/fimmu.2024.1457230 (PMC11461197; doi:10.3389/fimmu.2024.1457230)

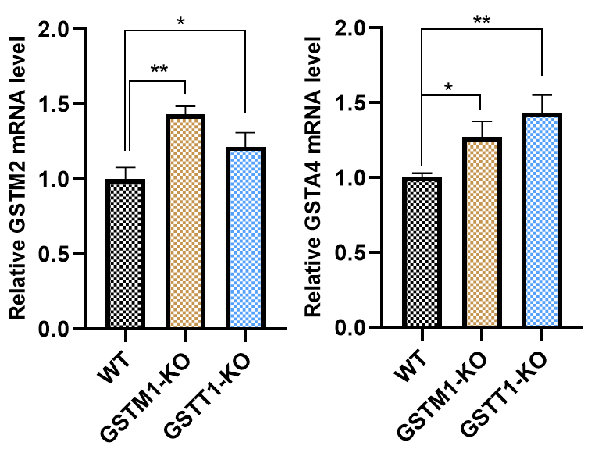

Supplement: Supplementary Figure 1 — The mRNA levels of GSTM2 and GSTA4 in WT, GSTM1-KO and GSTT1-KO mice. The data are presented as the mean ± SD, *P<0.5, **P<0.01, ***P<0.001, n=3. [file Image1.tif]

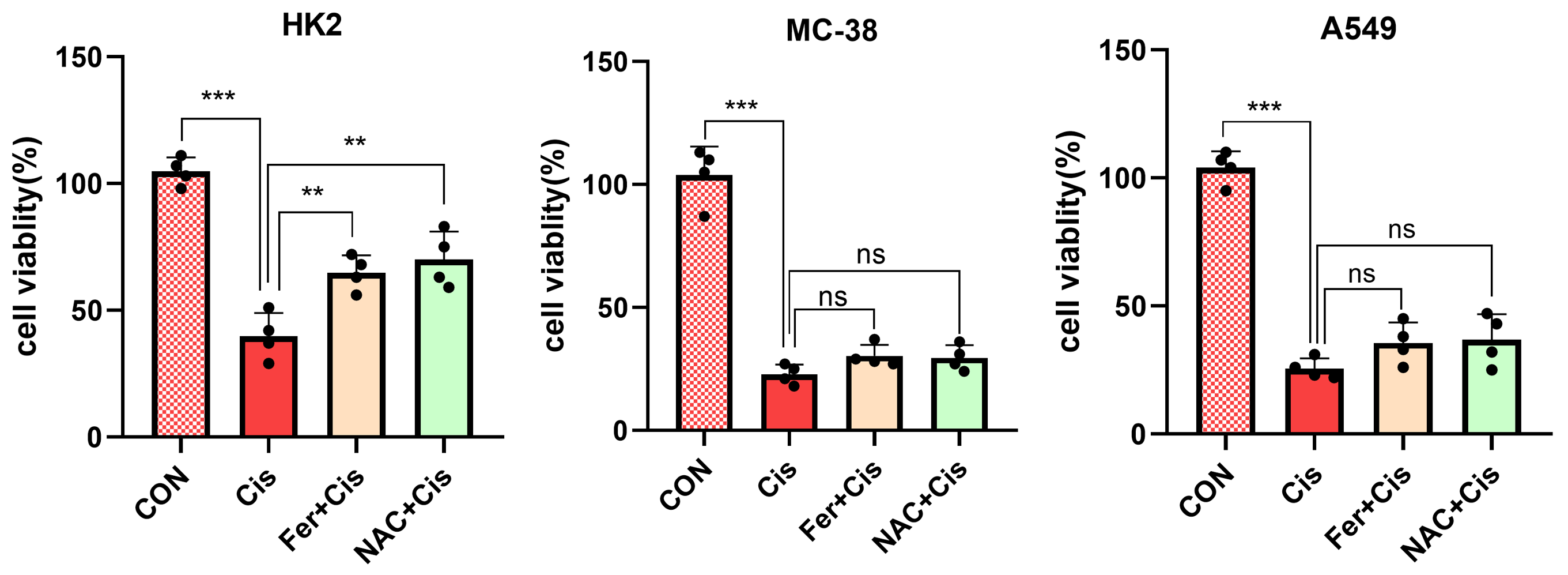

Supplement: Supplementary Figure 2 — The HK2, colon and lung cancer cell lines were pretreated with Fer-1/NAC and then treated with cisplatin for 24 h. Cell survival rate was detected by CCK8 assay. The data are presented as the mean ± SD, *P<0.5, **P<0.01, ***P<0.001, n=4. [file Image2.tif]

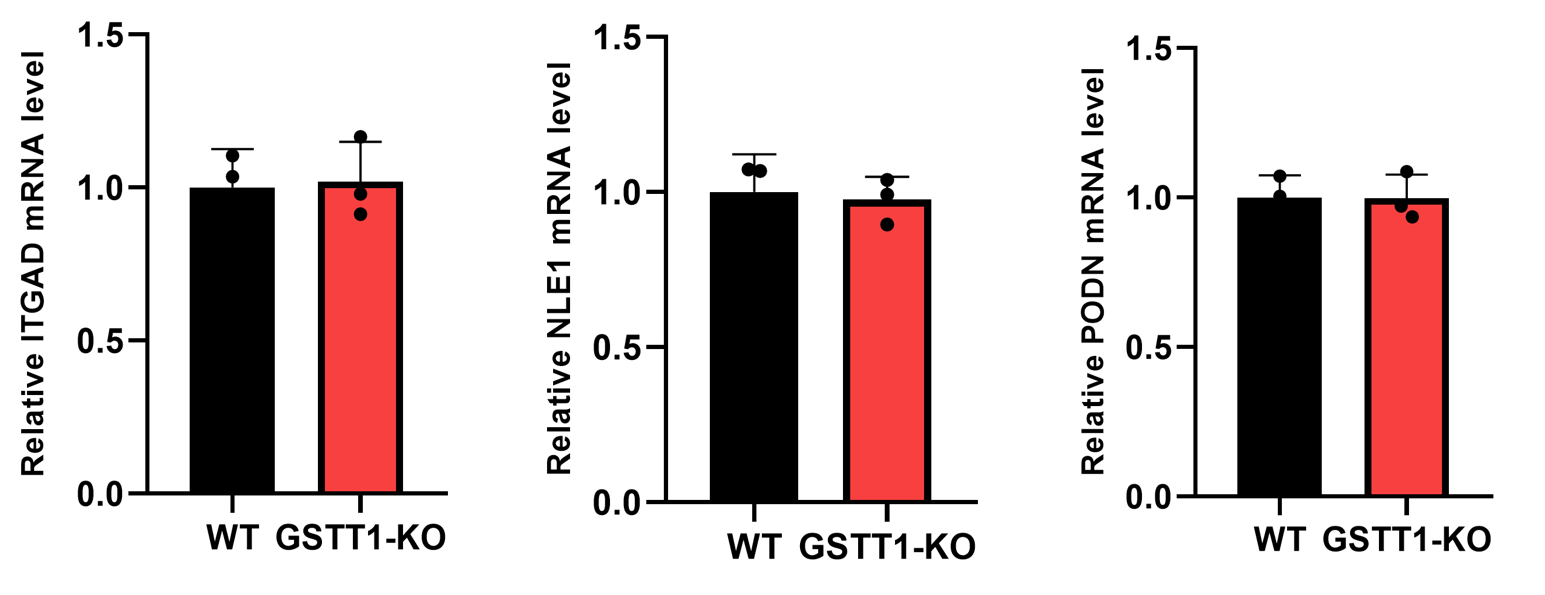

Supplement: Supplementary Figure 3 — The mRNA levels of ITGAD, NLE1 and PODN in WT and GSTT1-KO mice. The data are presented as the mean ± SD, *P<0.5, **P<0.01, ***P<0.001, n=3. [file Image3.tif]

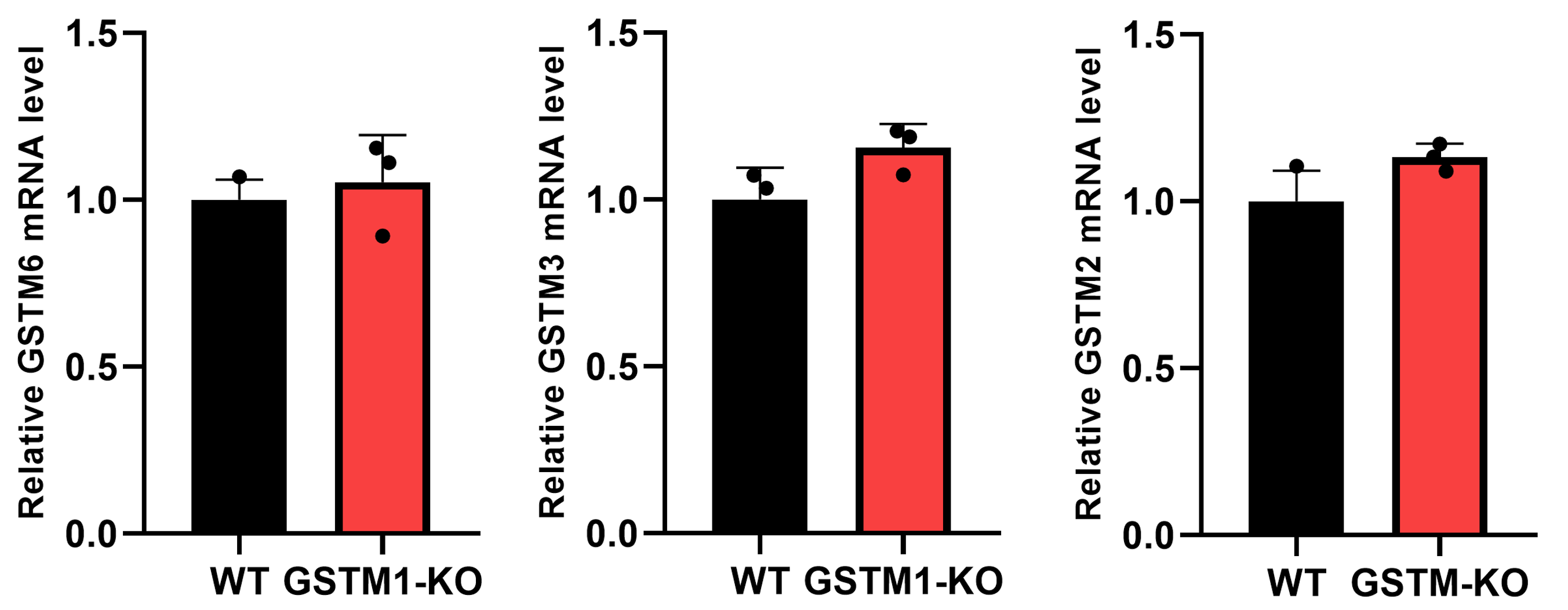

Supplement: Supplementary Figure 4 — The mRNA levels of GSTM6, GSTM3 and GSTM2 in WT and GSTM1-KO mice. The data are presented as the mean ± SD, *P<0.5, **P<0.01, ***P<0.001, n=3. [file Image4.tif]
